# Persistence of EBV Antigen-Specific CD8 T Cell Clonotypes during Homeostatic Immune Reconstitution in Cancer Patients

**DOI:** 10.1371/journal.pone.0078686

**Published:** 2013-10-25

**Authors:** Emanuela M. Iancu, Philippe O. Gannon, Julien Laurent, Bhawna Gupta, Pedro Romero, Olivier Michielin, Emanuela Romano, Daniel E. Speiser, Nathalie Rufer

**Affiliations:** 1 Department of Oncology, Lausanne University Hospital Center (CHUV) and University of Lausanne, Lausanne, Switzerland; 2 Ludwig Center for Cancer Research, University of Lausanne, Lausanne, Switzerland; New York University, United States of America

## Abstract

Persistent viruses are kept in check by specific lymphocytes. The clonal T cell receptor (TCR) repertoire against Epstein-Barr virus (EBV), once established following primary infection, exhibits a robust stability over time. However, the determinants contributing to this long-term persistence are still poorly characterized. Taking advantage of an *in vivo* clinical setting where lymphocyte homeostasis was transiently perturbed, we studied EBV antigen-specific CD8 T cells before and after non-myeloablative lympho-depleting chemotherapy of melanoma patients. Despite more advanced T cell differentiation, patients T cells showed clonal composition comparable to healthy individuals, sharing a preference for *TRBV20* and *TRBV29* gene segment usage and several co-dominant public TCR clonotypes. Moreover, our data revealed the presence of relatively few dominant EBV antigen-specific T cell clonotypes, which mostly persisted following transient lympho-depletion (TLD) and lymphocyte recovery, likely related to absence of EBV reactivation and *de novo* T cell priming in these patients. Interestingly, persisting clonotypes frequently co-expressed memory/homing-associated genes (*CD27*, *IL7R*, *EOMES, CD62L/SELL* and *CCR5*) supporting the notion that they are particularly important for long-lasting CD8 T cell responses. Nevertheless, the clonal composition of EBV-specific CD8 T cells was preserved over time with the presence of the same dominant clonotypes after non-myeloablative chemotherapy. The observed clonotype persistence demonstrates high robustness of CD8 T cell homeostasis and reconstitution.

## Introduction

Primary Epstein-Barr virus (EBV) infection is associated with massive viral replication. In spite of the generation of a robust T cell specific immune response, EBV establishes a latent infection in B cells [1]. Nevertheless, healthy EBV-infected individuals remain asymptomatic throughout their life due to viral containment by antigen-specific memory CD8 T lymphocytes [1-3]. As increasing attention is devoted to optimizing therapeutic vaccination strategies against cancer, the immune control of chronic EBV infection represents an interesting model system to study the mechanisms involved in the generation and maintenance of life-long protective immune responses.

The antigen-primed CD8 T cell pool is highly heterogeneous and comprises T cell subpopulations at varying degrees of cellular differentiation based on their phenotype, function and anatomic location, thus making the distinction between “effector” and “memory” cytolytic T cells challenging [4-6]. Generally, memory-like T cells display long-term survival and self-renewal abilities, high proliferative potential, and the capacity to rapidly and efficiently produce effector cells in response to antigen re-encounter [7-9]. In contrast, effector T cells have the capacity to migrate to the site of inflammation, to kill antigen-bearing target cells and secrete various cytokines (e.g. IFNγ, TNFα) [10].

Whether memory cell responses are maintained for prolonged periods or periodically “renewed” remains a matter of debate. It is a particularly difficult question to address in humans since it requires in-depth analysis of protective immune responses over extended periods of time. In this regard, the T cell receptor (TCR) is an excellent marker that allows antigen-specific responses to be followed along T cell differentiation and over time [11]. Thorough analyses of both viral and tumor antigen-specific CD8 T cell responses have revealed that the antigen-specific T cell repertoire is generally composed of highly frequent (also defined as dominant) as well as less frequent (defined as non-dominant) T cell clonotypes [12,13]. Through a process known as TCR clonotype selection, certain clonotypes may become more dominant along T cell differentiation [14-16] or throughout the time course of infection [17]. Persistence of human virus-specific T cell clonotypes over several years has been demonstrated in humans with various viral infections: influenza [18], herpes simplex virus [19,20], EBV [21], cytomegalovirus (CMV) [14] and human immunodeficiency virus (HIV) [22]. Recently, Klarenbeek and colleagues [23] addressed the question of whether the clonal repertoire that is established during the early phase of infections against CMV and EBV is maintained over extended periods of time. They found that immune responses were very stable, and once established did not evolve during the 5-year follow up [23]. While herpes virus-specific T cell responses revealed an unprecedented stability of the TCR clonotype repertoire over time following primary infection, less is known regarding their persistence during conditions of immunological stress in asymptomatic carriers.

In the present study, we examined the robustness of the EBV antigen-specific responses in an *in vivo* setting where the balance between virus and immune response may be temporarily compromised following transient lympho-depletion (TLD). Specifically, we evaluated the EBV antigen-specific CD8 T cell clonotype composition and persistence in melanoma patients who were treated with non-myeloablative chemotherapy regimen, followed by adoptive cell transfer (ACT) of autologous peripheral blood mononuclear cells (PBMCs) [24,25]. To quantitatively assess virus-specific T cell responses, direct *ex vivo* clonotypic analyses combined to gene expression profiling of individual antigen-specific T cells were performed [13]. The anti-viral T cell responses in patients were more differentiated compared with healthy individuals, comprising both memory and effector CD8 T cells. Dominant TCR beta-chain clonotypes, including several public TCR sequences, were found to persist with time in healthy individuals and following TLD and ACT among patients. We then studied T cell clonotypes with fluctuating frequencies following TLD and immune reconstitution, and observed that clonotypes with increased frequency carried a polyfunctional memory/homing gene expression profile (*CD27*, *IL7R*, *EOMES, CD62L/SELL* and *CCR5*). Altogether, our data suggest that the EBV-specific T cell repertoire persists not only under steady-state conditions but also during transient immunological perturbations.

## Materials and Methods

### Ethics statement

The clinical studies were designed and conducted according to the relevant regulatory standards, and approved by (i) the ethical commission of the University of Lausanne, (ii) the LICR Protocol Review Committee, and (iii) the Swiss national regulatory authority (Swissmedic). They have been registered in the NCI clinical trials under the number NCT00324623 and EU20607 (www.clinicaltrials.gov). All blood samples from patients were collected upon written informed consent. Peripheral blood samples from four EBV-positive healthy donors BCL3, BCL4, BCL7 and BCL8, aged between 25 and 45 years, were collected at two time-points, in the years 2002 and 2006, and all donors gave written informed consent.

### Melanoma patients and treatment

Blood samples from five EBV-positive melanoma patients, aged between 39 to 75 years, enrolled in phase I clinical trials at the Department of Oncology of the Lausanne University Hospital (Switzerland), were taken at various time-points before and after treatment. After collection of PBMCs by leukapheresis (Leuka), patients received non-myeloablative chemotherapy regimen consisting of either busulfan at 2 mg/kg (patients LAU 618 and LAU 672) or cyclophosphamide (CTX) at 30 mg/kg (LAU 1144 and first round for LAU 1013) or 60 mg/kg (LAU 936 and second round for LAU 1013) for two days and fludarabine at 30 mg/m^2^ for 3 days [24,25]. Three days after the last injection of fludarabine, autologous PBMCs were reinfused and peptide vaccination with the Melan-A analog peptide emulsified in incomplete Freunds’ adjuvant was given subcutaneously as previously described [24]. Patient LAU 1144 also received a soluble LAG-3 protein as adjuvant. 

### Antibody levels against EBV in melanoma patients

Plasma samples were taken from all patients at several time-points before and after lympho-depleting treatment and analyzed for alterations in antibody levels known to be associated with a state of EBV reactivation. Specifically, we compared the plasma antibody levels against three EBV expressed proteins: EBNA-IgG (Epstein-Barr Nuclear Antigen), EA-IgG (Early Antigen), VCA-IgG and IgM (Viral Capsid Antigen). EBV-specific antibodies were assayed using the AtheNA Multi-Lyte EBV test system (Zeus scientific, Sommerville, NJ, USA) on a Luminex 200 reader according to the manufacturer instructions. Results were expressed as arbitrary units.

### Cell preparation and flow cytometry

PBMCs were obtained by density centrifugation using Ficoll-Hypaque (Pharmacia, Uppsala, Sweden). CD8 T lymphocytes were positively enriched from cryopreserved PBMCs using anti-CD8-coated magnetic microbeads (Miltenyi Biotech, Bergish Gladbach, Germany). PE-labeled HLA-A*0201/peptide multimers were prepared as described previously [26] with the HLA-A2 restricted epitope GLCTLVAML (referred as A2/GLC) derived from the EBV lytic protein BMFL1. Cells were first stained with PE-labeled multimers for 1hr at room temperature (RT) in PBS, 0.2% BSA, 50 uM EDTA, and then with appropriate antibodies (20 min at 4°C). Cells were either directly analyzed (LSR-II flow cytometer; BD Biosciences, San Diego, CA) or sorted into defined populations using a FACSVantage SE machine (BD Biosciences). The following monoclonal Abs were purchased from BD Biosciences or BD PharMingen; anti-CD28-FITC, anti-CD8-allophycocyanin/Cy7, anti-CCR7 rat IgG mAB, goat anti-rat IgG-allophycocyanin, and CD3-AmCyan-A. Anti-CD45RA-PE-Texas Red mAb was purchased from Beckman Coulter (Marseille, France).

### Generation of T cell clones and culture

HLA-A2/multimer^+^ CD8^+^ T cell subsets were defined as effector-memory (EM) CCR7^-^CD45RA^-^CD28^+^ (EM28^pos^), CCR7^-^CD45RA^-^CD28^-^ (EM28^neg^) and effector CCR7^-^CD45RA^+^CD28^-^ (EMRA), and were sorted by flow cytometry directly *ex vivo* (Figure S1). Sorted cells were cloned by limiting dilution and expanded in RPMI 1640 medium supplemented with 8% human serum (HS), 150 U/ml recombinant human IL-2 (rhIL-2; a gift from GlaxoSmithKline), 1 microgram/ml phytohemagglutinin (PHA; Sodiag, Losone, Switzerland) and 1x10^6^/ml irradiated allogeneic PBMCs (3’000 rad) as feeder cells. A2/multimer^+^ T cell clones were expanded by periodic (every 15 days) restimulation in 24-well plates with PHA, irradiated feeder cells and hrIL-2. 

### Direct ex vivo cell sorting, cDNA amplification and single cell gene-specific PCR

Single or five-cell aliquots were sorted directly *ex vivo* from T cell subsets of interest and cDNA preparation and global cDNA amplification performed as previously described [27,28]. Gene signature of individual T cell was identified by gene-specific PCRs as described [28] and PCR products visualized after electrophoresis on a 2.5% agarose gel. We used the following primers: *GAPDH*: 5’-GGACCTGACCTGCCGTCTAG-3’; rev-5’-CCACCACCCTGTTGCTGTAG-3’, *beta2 microglobulin*: 5’-CCAGCAGAGAATGGAAA GTC-3’; rev-5’-GATGCTGCTTACATGTCTCG-3’, *CCR7*: 5’-CCAGGCCTTATCTCC AAGACC-3’; rev-5’-GCATGTCATCCCCACTCTG-3’, *CD27*: 5’-ACGTGACAGAGTGCC TTTTCG-3’; rev-5’-TTTGCCCGTCTTGTAGCATG-3’, *IL7R* (IL-7Ra/CD127): 5’-ATC TTGGCCTGTGTGTTATGG-3’; rev-5’-ATTCTTCTAGTTGCTGAGGAAACG-3’; *EOMES* (eomesodermin): 5’-AGCAGGCTGTGAACATTGG-3’; rev-5’-TTGACTCCTGGG CCTAGTATC-3’, *CCR5*: 5-TCAGCAGGAAGCAACGAAGG-3’; rev-5’-TCTTTGACTTG GCCCAGAGG-3’, *KLRD1* (CD94): 5’-GTGGGAGAATGGCTCTG CAC-3’; rev-5’-TGAGCTGTTGCTTACAGATATAACGA-3’, *IFNG* (IFN-): 5’-GCCAAC CTAAGCAAGATCCCA-3’; rev-5’-GGAAGCACCAGGCATGAAATC-3’, *PRF1* (Perforin): 5’-TTCACTGCCACGGATGCCTAT-3’; rev-5’-GCGGAATTTTAGGTGGCCA-3’, *GZMB* (Granzyme B): 5’-GCAGGAAGATCGAAAGTGCGA-3’; rev-5’-GCATGCCAT TGTTTCGTCCAT-3’, *SELL* (CD62L): 5’-CCGTCTGTGAATTGGACCAT-3’; rev-5’-AAC AGCAAAACCCCCAAACT-3’. 

### TCR spectratyping, sequencing and TCR clonotyping

TCR spectratyping [14] was used to identify all TCR clonotypes, which were classified according to the ImMunoGeneTics (IMGT) nomenclature proposed by Lefranc and colleagues (http://imgt.org/)[29]. To rapidly identify TRBV segment usage, cDNA pools of EBV antigen-specific CD8 T cells were initially subjected to individual PCR using a set of previously validated fluorescent-labeled forward primers specific for the 22 TCR *BV* subfamilies and one unlabeled reverse primer specific for the constant region of the beta chain of the TCR [30]. This TRBV-CDR3 spectratyping analysis represents a prescreening step that allows saving time and reagents (data not shown). Once positive TRBV subfamilies were identified, individual cDNA samples generated from either *ex vivo* sorted single cell samples (n = 477) and 5-cell samples (representing the equivalent of 300 to 450 EBV-specific CD8 T cells per healthy donor or melanoma patient) or from *in vitro* generated T cell clones (healthy donors, n = 530 clones; melanoma patients, n = 779 clones) were subjected to TRBV-specific PCRs. Separation and detection of amplified PCR fragments that contained the entire CDR3 segment were performed in the presence of fluorescent size markers on an ABI PRISM 310 Genetic Analyzer (AppliedBiosystems/Life Technologies Corporation, Zug, Switzerland) and data were analyzed with GeneScan 3.7.1 (AppliedBiosystems). In the last step, PCR products of interest were directly purified and sequenced with the reverse primer (Fasteris SA, Geneva, Switzerland). The majority of PCR products were sequenced, however for several dominant TCR clonotypes (n = 8 for HDs; n = 10 for patients), unique primers corresponding to the *CDR3* gene segment were designed and used for clonotyping PCRs as previously described [15]. All *ex vivo* single cell, 5-cell, and *in vitro* generated T cell clone cDNA samples from healthy donors and melanoma patients were processed in the same rigorous approach.

### Statistical analyses

As indicated throughout the text, Kruskal-Wallis non-parametric, one-way ANOVA and Spearman’s correlations were performed with Prism 5.0 (La Jolla, California, USA) and *P* < 0.05 was considered as statistically significant. Co-expression pie charts were compared with each other using 10’000 permutations calculated with the Software SPICE 5.2 (NIH, Bethesda, USA). 

## Results

### Enhanced effector cell differentiation of EBV antigen-specific CD8 T cells following transient lympho-depletion and immune reconstitution

**Figure 1:**
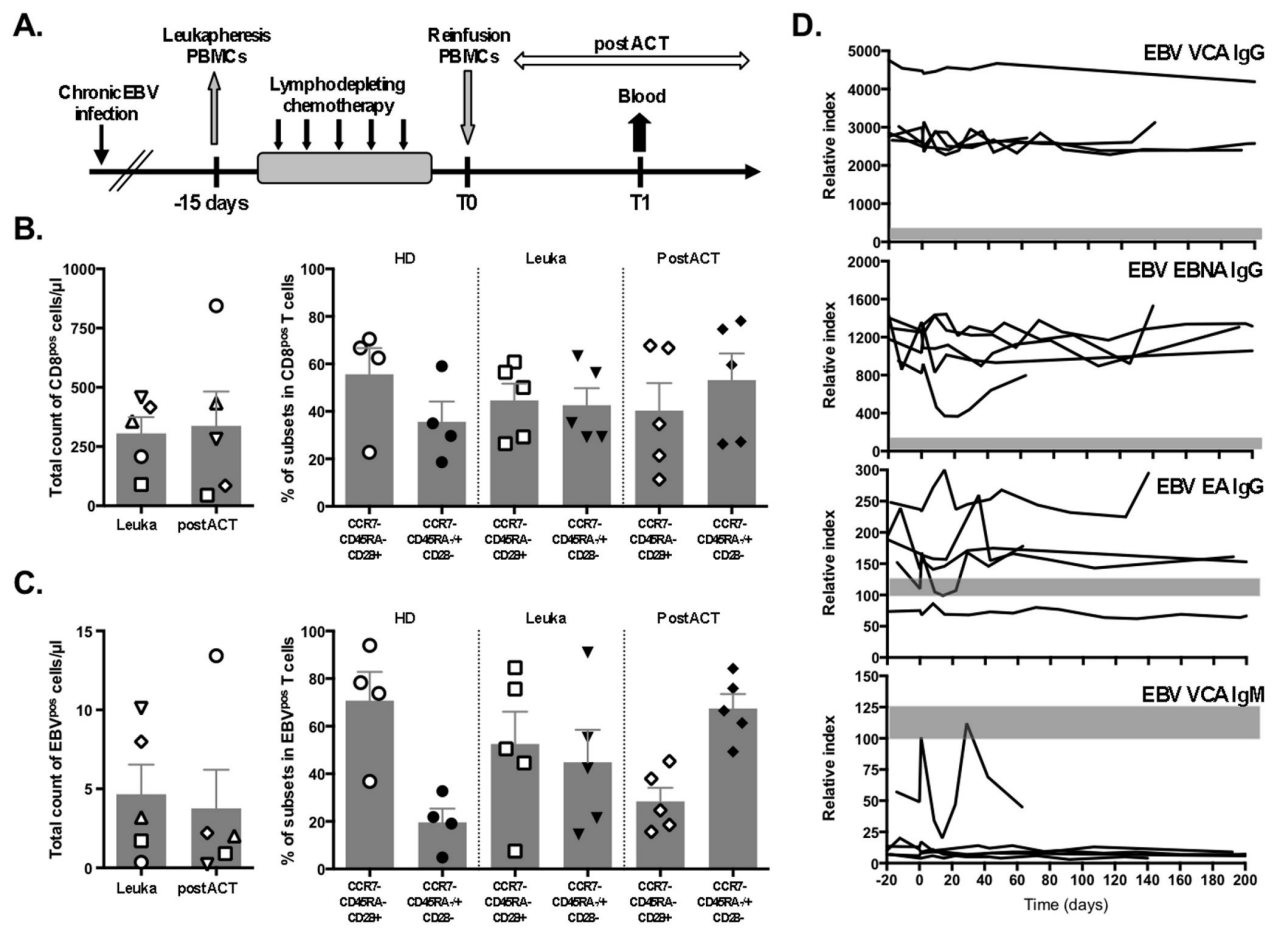
Direct *ex* *vivo* analysis of HLA-A*0201/BMFL1 specific CD8 T cells from healthy donors and melanoma patients. **A**. Schematic representation of non-myeloablating lympho-depleting treatment followed by ACT of PBMCs for melanoma patients. Blood samples were analyzed for all patients at the leukapheresis time-point (Leuka) before TLD chemotherapy and post-ACT (time-point T1), which corresponded to day 32 (patient LAU 1144), day 45 (LAU 1013), or day 60 (LAU 618, LAU 936, and LAU 672). **B**. Left panel; total counts of CD8 T cells from five melanoma patients at Leuka and post-ACT. Right panel; phenotype of total CD8 T cells showing proportions of early-differentiated (EM28^pos^; CCR7^neg^CD45RA^neg^CD28^pos^) and late-differentiated (comprising of EM28^neg^; CCR7^neg^CD45RA^neg^CD28^neg^ and EMRA; CCR7^neg^CD45RA^pos^CD28^neg^) subsets from four healthy donors (HDs) and from five melanoma patients at Leuka and post-ACT. **C**. Left panel; total counts of EBV-specific CD8 T cells in peripheral blood from five melanoma patients at Leuka and post-ACT time points. Right panel; phenotype of EBV-specific CD8 T cells showing proportions of early-differentiated (EM28^pos^; CCR7^neg^CD45RA^neg^CD28^pos^) and late-differentiated (comprising of EM28^neg^; CCR7^neg^CD45RA^neg^CD28^neg^ and EMRA; CCR7^neg^CD45RA^pos^CD28^neg^) subsets from four healthy donors and from five melanoma patients at Leuka and post-ACT. **D**. EBV-specific antibody levels measured in plasma samples from five melanoma patients at various time-points before and after lympho-depleting treatment. Day 0 on the x-axis represents the day of ACT. Results are represented as arbitrary units on a relative index scale. Plasma level evolution is shown separately for each anti-EBV antibody (anti-VCA IgM and IgG, anti-EBNA IgG and anti-EA IgG), and the grey bar represents the cut-off between negative and positive status for each marker.

Recent immunotherapy trials have shown that lympho-depletion induced by non-myeloablative chemotherapy favored subsequent expansion of adoptively transferred T cells by homeostatic mechanisms ([31]; Figure 1A). To further refine this strategy, we had previously analyzed the impact of three different non-myeloablative conditioning chemotherapy regimens on lympho-depletion and reconstitution in melanoma patients [24,25]. The three chemotherapeutic regimens induced significant transient depletion of lymphocytes, followed by efficient recovery of total lymphocyte [24,25] and CD8 T cell (Figure 1B, left panel) counts to normal levels four weeks after adoptive cell transfer (post-ACT). 

We then analyzed the CD8 T cells specific for the EBV lytic protein BMFL1 from five patients with stage IV melanoma before and after TLD (Figure S1) and from four healthy donors. The overall proportion of EBV antigen-specific T cells before and after treatment remained unchanged (Figure 1C; left panel; *P* = 0.625, Kruskal-Wallis). As compared to healthy individuals, BMFL1-specific CD8 T cells from melanoma patients showed more advanced effector cell differentiation with increased percentages of EM28^neg^ (defined as CCR7^neg^CD45RA^neg^CD28^neg^) and EMRA (defined as CCR7^neg^CD45RA^pos^CD28^neg^) subsets already before treatment (i.e. at the time of leukapheresis) (Figure 1C; right panel). These subsets increased further and became dominant post-ACT. In healthy donors, such advanced effector cell differentiation is not typically observed within EBV-specific CD8 T cells, which resembles more closely to CMV-specific T cell responses [14,32]. To a weaker extent, advanced effector cell differentiation was also observed in the total CD8 T cell pool (Figure 1B; right panel), suggesting that preceding histories of tumor progression and received treatments, such as chemotherapy and immunotherapy, may have affected the CD8 T cell compartment.

### Transient lympho-depletion does not result in EBV reactivation

To examine the ability of the EBV antigen-specific CD8 T cell response to control viral activity during situations of diminished immune control, we determined whether non-myeloablative chemotherapy might have resulted in EBV reactivation. Plasma antibody levels for viral capsid antigen (VCA) IgG and IgM, Epstein-Barr nuclear antigen (EBNA) IgG, and early antigen (EA) IgG were measured before and after TLD and ACT treatments (Figure 1D)[33]. EBV-specific antibodies remained stable for several months after treatment in all patients. This is consistent with a latent EBV infection without viral reactivation, although the latter cannot be completely excluded. The EBV DNA copy numbers per million PBMCs were below the level of detection by real-time PCR reaction in blood samples before and after TLD (data not shown). 

### The ex vivo EBV antigen-specific CD8 T cell repertoire following transient lympho-depletion reveals clonotype persistence despite frequency fluctuations

We previously developed a strategy of global cDNA amplification at the 5-cell level suitable for the direct *ex vivo* assessment of individual TCR BV-CDR3beta (TRBV) gene segment usage [14,34]. Briefly, cDNA pools of EBV epitope-specific CD8 T cells were initially generated and used as a screen for recurrent TRBV families. Subsequently, the dominance of each TRBV family was determined by testing every single 5-cell sample, comprising the pool for the positive TRBV families, and representing the equivalent of 300 to 450 virus-specific CD8 T cells per individual. The amplified TRBV-CDR3-JB gene segments were ultimately sequenced or PCR-clonotyped to identify the unique TCR signature of each T cell clonotype, as described in Materials and Methods. Using this approach, we initially compared the TCR clonotype repertoire of directly *ex vivo* sorted 5-cell of EBV-specific T cells with that of single T cells generated by *in vitro* limiting dilution cultures. Comparable proportions of individual TCR clonotype signatures were found when using the *ex vivo* sorted 5-cell and the *in vitro* T cell cloning approaches (Figure S2A), in agreement with previous studies [14,15,28]. Moreover, independently performed *ex vivo* sorting experiments on single virus antigen-specific T cells, revealed similar relative frequencies of dominant T cell clonotypes (Figure S2B). Altogether, these results indicate that our direct *ex vivo* approach allows the high-resolution molecular characterization of the clonotype repertoire *in vivo* in well-defined antigen-specific subpopulations. 

**Figure 2:**
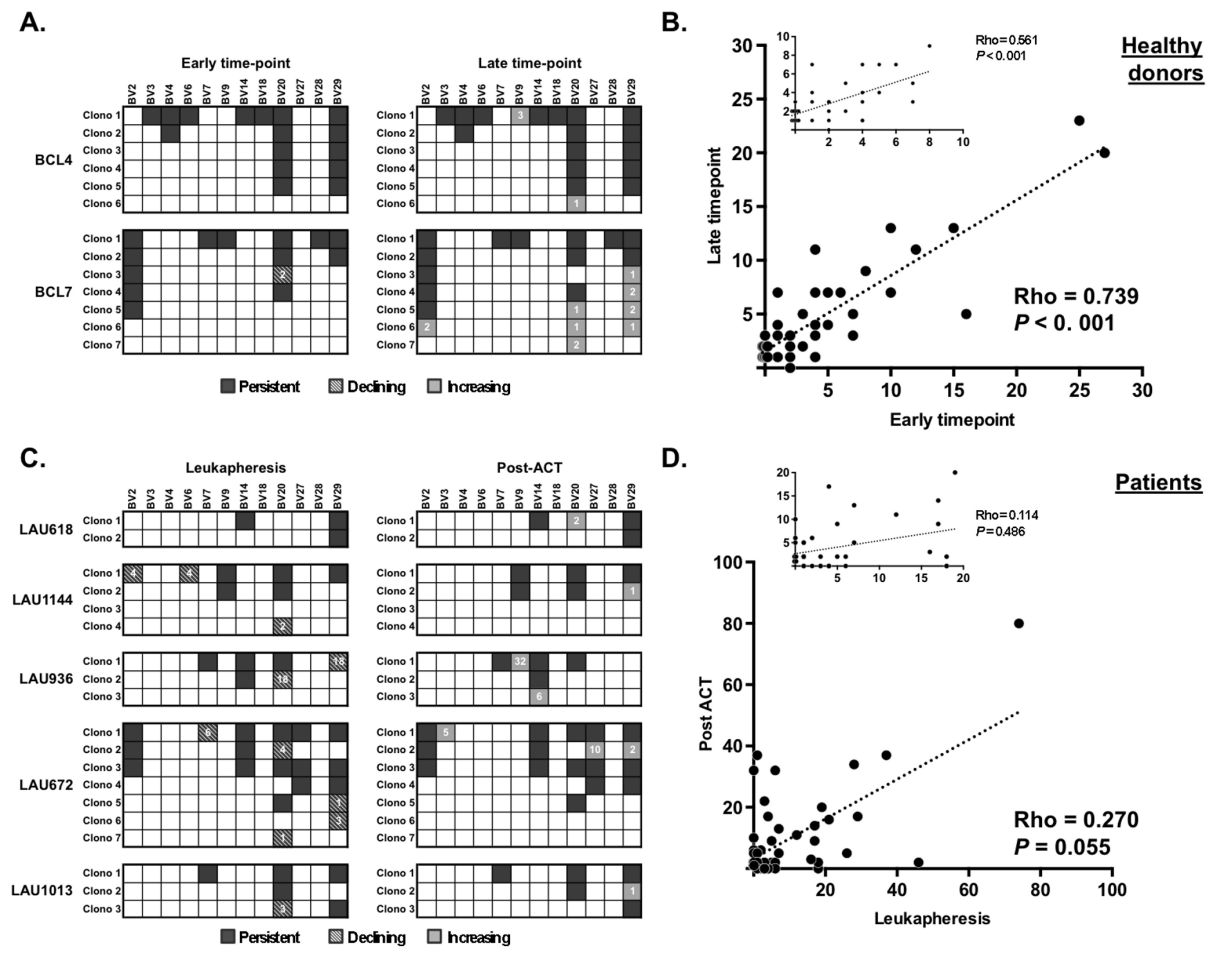
*Ex* *vivo* TCR beta-chain clonotype composition among EBV-specific CD8 T cells over time in healthy donors and melanoma patients. **A** and **C**. TRBV clonotype composition of EBV-specific CD8 T cells in PBMCs from two healthy donors (**A**) at early (2002) and late (2006) time-points, and in PBMCs from four melanoma patients (**C**) at Leuka time-point and post-ACT. Each clonotype is represented by its specific TRBV family and its code number (clono). Clonotype frequencies are calculated by the presence of each clonotypic sequence among individual 5-cell samples (healthy donors, n = 142; melanoma patients, n = 344) sorted directly *ex* *vivo*. Of note, only frequencies from either new “increasing” or “declining” clonotypes are depicted. **B and D**. Correlation of individual clonotype frequencies obtained from *ex* *vivo* sorted 5-cell samples (**B**) between blood samples at early (2002) and late (2006) time-points from two healthy donors and (**D**) between Leuka and post-ACT from five melanoma patients (Spearman’s correlation). Insets show the correlation between TCR clonotypes with frequencies below 10% for healthy donors and 20% for patients.

We next determined the persistence of EBV BMFL1-specific T cell clonotypes in healthy individuals over time. The majority of clonotypes were present at both early and late time-points (Figure 2A). Moreover, a correlation showed a statistically significant association between the frequencies of clonotypes detected over a period of 4 years (Figure 2B; Rho = 0.739, *P* < 0.001, Spearman’s correlation). We could observe the appearance of new clonotypes over time, however these newly detected clonotypes were of low frequencies (

< 5%) (Figure 2B). Together, these results are in line with those previously reported by our group [14] and others [23], and indicate that once established in healthy adults, the clonal composition during chronic herpes viruses infection is kept stable for at least several years.

We further assessed the EBV antigen-specific TCR repertoire in five melanoma patients to determine the recovery potential of dominant T cell clonotypes following TLD and ACT (Figure 2C). Similarly to the healthy donors, the overall TCR repertoire persisted following TLD and ACT with the large majority of clonotypes being detectable before and after treatment (Figure 2C). Except for patient LAU 936, the clonotypes that either disappeared or appeared were of low frequency (<5%), which may be related to the sensitivity of the technique more than the actual appearance or disappearance of a specific clonotype. Nonetheless, we found more pronounced fluctuations in the frequencies of the detectable clonotypes among patients compared with healthy donors, which was especially evident for the clonotypes with frequencies <20% (Figure 2D). Collectively, our data show that the clonal composition of EBV-specific CD8 T cells was globally maintained in melanoma patients undergoing TLD and followed by immune reconstitution, despite fluctuations in frequencies within specific patients and for clonotypes detectable at lower frequencies.

### EBV antigen-specific clonotypes bearing public TRBV sequences are frequently shared between healthy donors and melanoma patients

**Figure 3:**
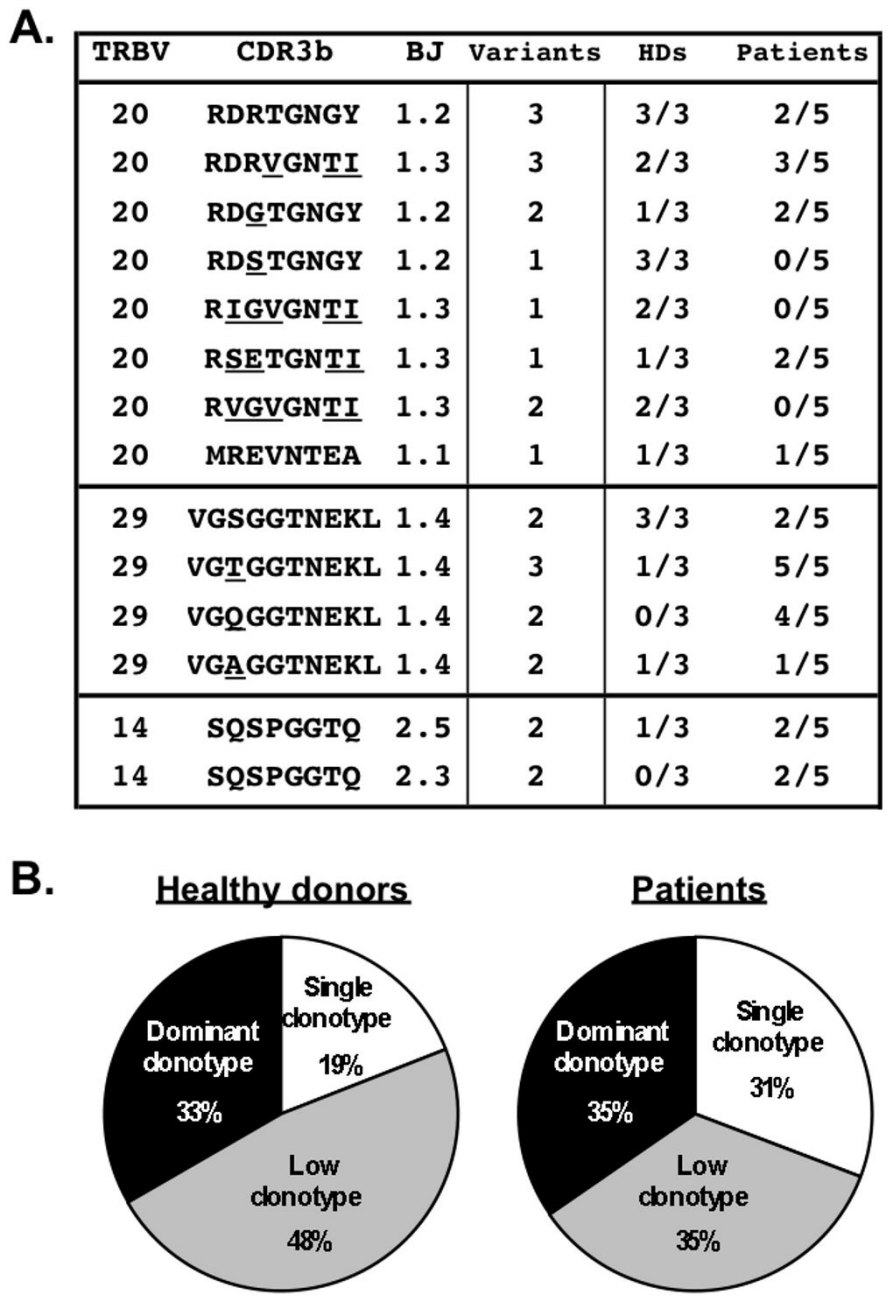
*In* *vivo* selection of public TCR clonotypes in healthy donors and melanoma patients. **A**. Compilation of the 14 public amino acid TCR beta-domain sequences detected among three healthy individuals and five melanoma patients. Each TCR beta-chain clonotype is described by the TRBV segment, CDR3-beta sequence, TRBJ segment, number of nucleotide sequence variants identified for each amino acid sequence, as well as the proportion of healthy donors and patients from which each sequence was identified. **B**. Distribution of public sequences within the overall EBV-specific CD8 T cells in healthy donors and patients according to their relative frequency; dominant clonotypes (with frequency >5%) are represented in black, low dominant (1-5%) in grey and unique sequences in white.

A major similarity of EBV epitope-specific CD8 T cells between healthy adults and patients was the preferential *in vivo* selection of dominant clonotypes with TRBV chains belonging to the *TRBV14*, *TRBV20* and *TRBV29* families (Figure 2). We also identified at least fourteen public TRBV sequences, with two CDR3 motifs RDxTGNGY and VGxGGTNEKL, which were over-represented and shared within healthy individuals and melanoma patients (Figure 3A). Public clonotypes are defined by the presence of the same identical TRBV-CDR3-BJ sequence found in at least two unrelated individuals. In line with previous reports [35], several of the public TRBV clonotypes identified in the present study were encoded by two or three different nucleotide sequence variations. When we compared the frequencies of public TRBV clonotypes within the overall EBV-specific CD8 T cell responses, a third of them represented dominant clonotypes (with frequencies >5%) (Figure 3B). Between 35 to 48% of EBV antigen-specific clonotypes were found at low frequencies (between 1 and 5%), while about one quarter were found only once in healthy donors or patients. Together, our data showed evidence for a high level of similarity of EBV BMFL1-specific CD8 T cells between melanoma patients and healthy individuals, as illustrated by (i) the sharing of frequent public TRBV sequences and (ii) the persistence of these public TRBV clonotypes with time and following TLD.

### Enhanced gene expression polyfunctionality by individual EBV antigen-specific CD8 T cells after transient lympho-depletion and immune reconstitution

**Figure 4:**
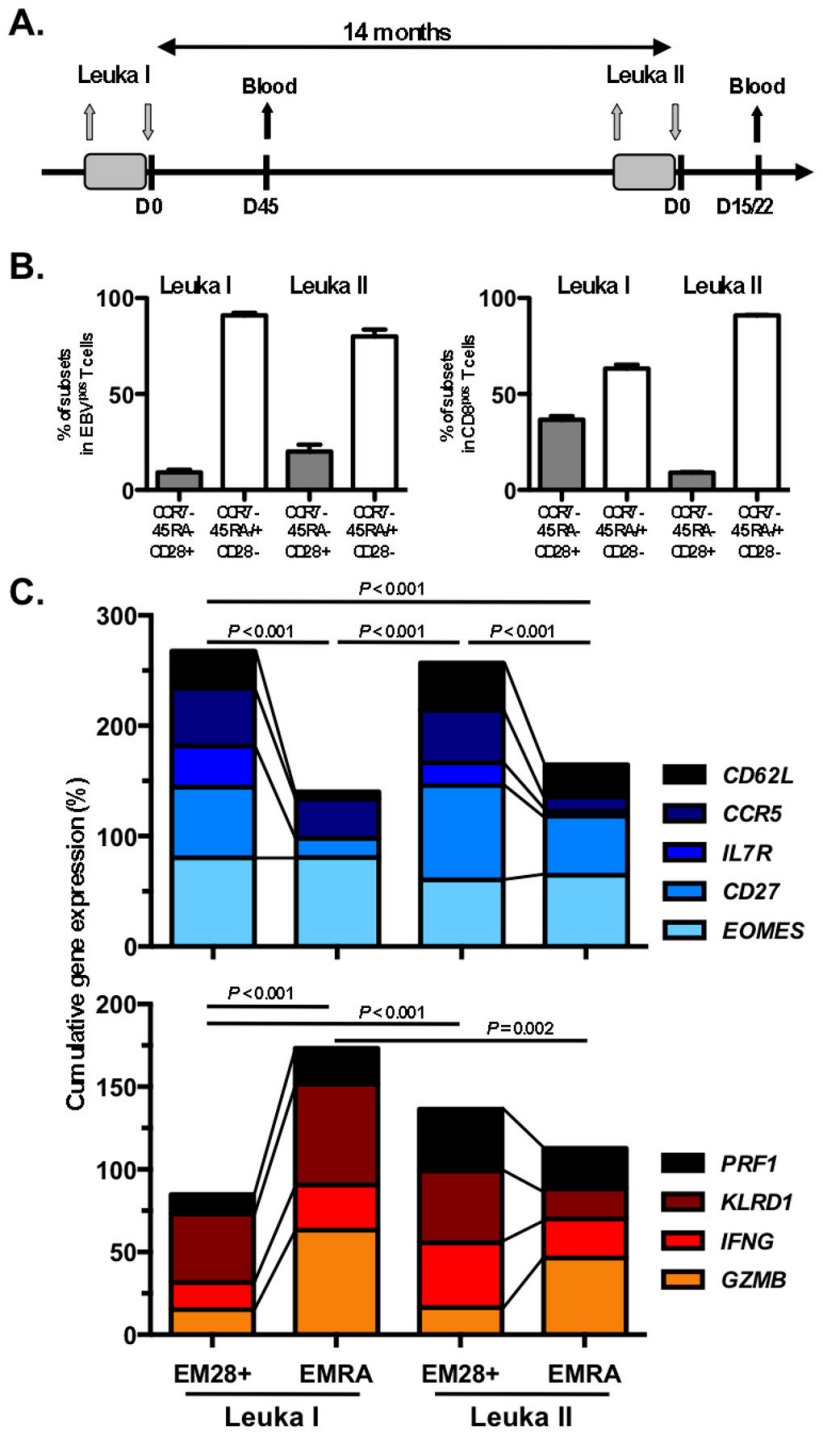
*Ex* *vivo* gene-expression profiling of single EBV antigen-specific CD8 T cells following cell differentiation from patient LAU 1013. **A**. Schedule of the treatment regimen for patient LAU 1013, who received two rounds of TLD (depicted as grey boxes). The grey arrows indicate leukapheresis (Leuka I and Leuka II) and reinfusion of PBMCs, respectively. Blood samples were taken at day 45 (post-Leuka I) and at day 15/22 (post-Leuka II) after reinfusion. **B**. Phenotype of EBV BMFL1-specific CD8 T cells (left panel) and total CD8 T cells (right panel) showing the proportions of early-differentiated (EM28^pos^; CCR7^neg^CD45RA^neg^CD28^pos^) and late-differentiated (comprising of EM28^neg^; CCR7^neg^CD45RA^neg^CD28^neg^ and EMRA; CCR7^neg^CD45RA^pos^CD28^neg^) subsets in PBMCs taken from patient LAU 1013 at Leuka I (n = 2) and Leuka II (n = 3) time points. Error bars (mean +/- SD) represent independent experimental replicates. **C**. Direct *ex* *vivo* cumulative expression of memory/homing-associated genes (*CD62L/SELL*, *CCR5*, *IL7R*, *CD27* and *EOMES*) and effector-related genes (*PRF1*, *KLRD1/CD94*, *IFNG* and *GZMB*). Single EBV-specific CD8 T cells were sorted from early-differentiated EM28^pos^ and late-differentiated EMRA at Leuka I (n = 266) and Leuka II (n = 162) time-points and processed for global cDNA amplification as described in Materials and Methods. *P*-values were performed by one-way ANOVA test; ns, not significant.

To further investigate the effect of TLD and ACT, we characterized the evolution of the EBV epitope-specific CD8 T cell response in patient LAU 1013 who underwent two successive cycles of TLD and immune reconstitution (Figure 4A), without showing signs of EBV reactivation (Figure 1D). The frequency of EBV-specific early-differentiated “memory-like” EM28^pos^ and late-differentiated EM28^neg^/EMRA CD8 T cells were comparable between the two leukapheresis time-points (Leuka I and Leuka II) (Figure 4B). In line with the data shown in Figure 1, we observed enhanced effector cell differentiation of the EBV-specific T cells before (at Leuka I) and following lympho-depletion (Leuka II), when compared to virus-specific T cells from healthy donors. 

Recently, we optimized the previously described strategy of global cDNA amplification for the direct *ex vivo* single cell gene-expression profiling [13,28] and applied it here with the aim of determining the fine changes in gene expression signatures over the course of TLD and ACT treatment in individual T cells. Gene-specific PCR was done for multiple memory/homing-associated molecules (*CD27*, *IL7R*, *EOMES, CD62L/SELL* and *CCR5*), as well as effector-related genes known to be upregulated along T cell differentiation (*PRF1, KLRD1, IFNG* and *GZMB*) (Figure 4C). The highest overall expression of memory-associated genes was found at Leuka I in the early-differentiated EM28^pos^ virus-specific subset, whereas the equivalent late-differentiated EMRA T cells exhibited the greatest expression of effector-related genes. Importantly, EM28^pos^ virus-specific T cells at Leuka II showed enhanced expression of effector-associated mRNA transcripts compared to EM28^pos^ found after Leuka I (*P* < 0.001, ANOVA) (Figure 4C). 

**Figure 5:**
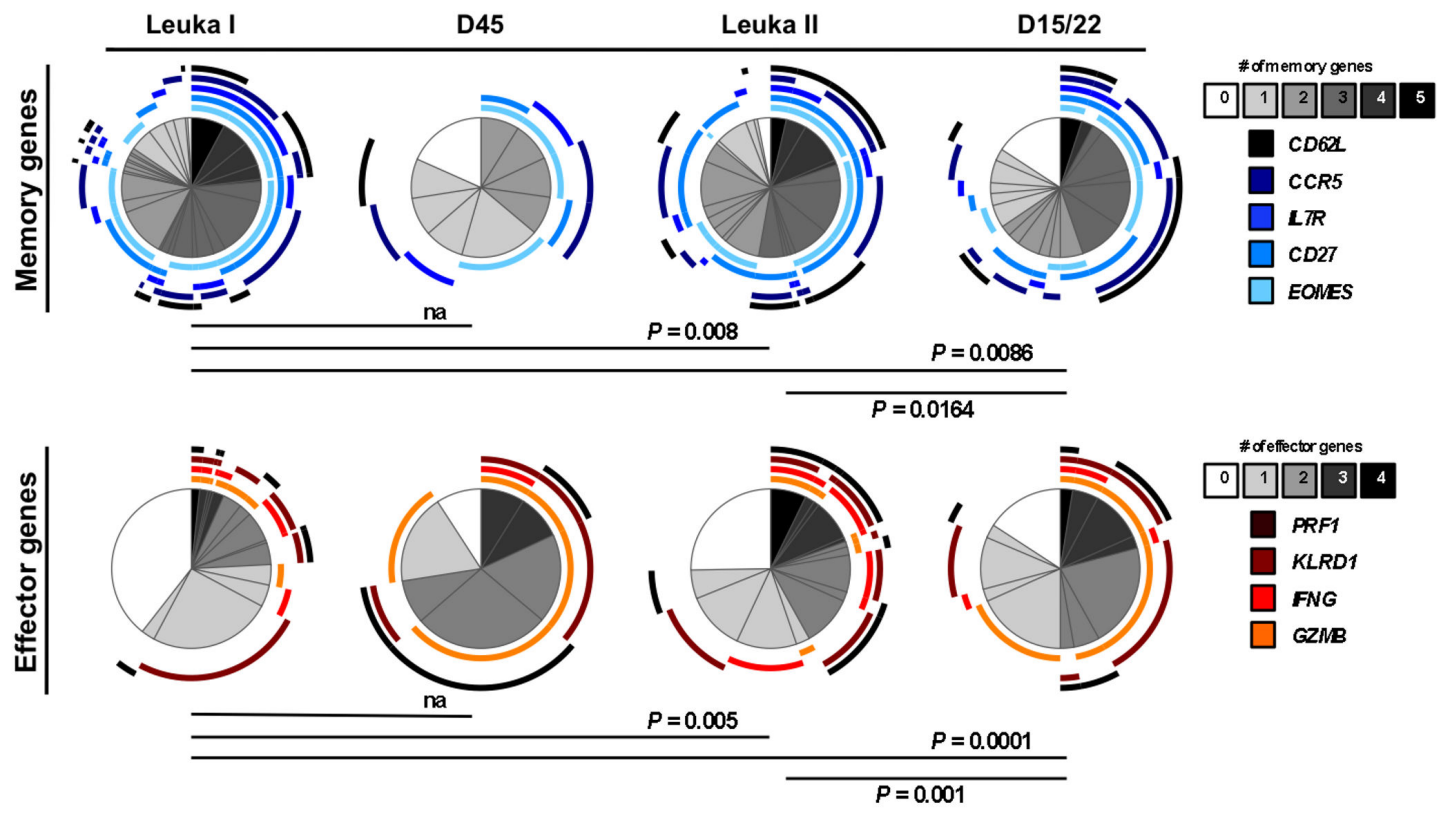
Co-expression of memory- and effector-related genes by individual EBV antigen-specific CD8 T cells following two rounds of TLD and immune reconstitution. *Ex* *vivo* gene expression polyfunctionality was determined as a measure of co-expression of the five memory/homing-associated and the four effector-associated gene transcripts in single cell samples from the EM28^pos^ subset at the indicated time-points (n = 332). Colors of the pie arcs depict the co-expression of individual memory or effector genes, whereas the color in the pie depicts the number of co-expressed memory- or effector-associated genes, as determined by SPICE 5.2. Increased polyfunctional gene co-expression (from 0 to 4 or 5) is shown as progressive grey gradients (from white to black). *P*-values of the permutation test are shown. Of note, at D45 (post-Leuka I) and D15/22 (post-Leuka II), EBV antigen-specific T cells were not sufficiently frequent to allow separation into defined subsets, thus data represent individual virus-specific T cells sorted from the total BMFL1/tetramer-specific population.

We then analyzed the level of co-expression patterns of either memory/homing-associated or effector-associated genes in individual EBV antigen-specific T cells from the early-differentiated EM28^pos^ subsets (Figure 5). T cells from Leuka I showed the largest proportion of cells co-expressing at least four memory-associated genes, followed by the virus-specific T cells isolated from the Leuka II EM28^pos^ and D15/22 subsets (Figure 5, top pies). Interestingly, the EM28^pos^ specific T cells from Leuka I depicted again limited co-expression of effector genes (Figure 5, bottom pies), contrasting with the increased gene expression diversity observed in both memory- and effector-associated genes following the second round of lympho-depletion (Leuka II and D15/22). These findings further support the notion that TLD and immune recovery induced T cell differentiation of EBV-specific CD8 T cells, while maintaining memory-related gene co-expression to a relative high level. 

### In vivo persistent EBV antigen-specific T cell clonotypes reveal a polyfunctional memory gene-expression profile

**Figure 6:**
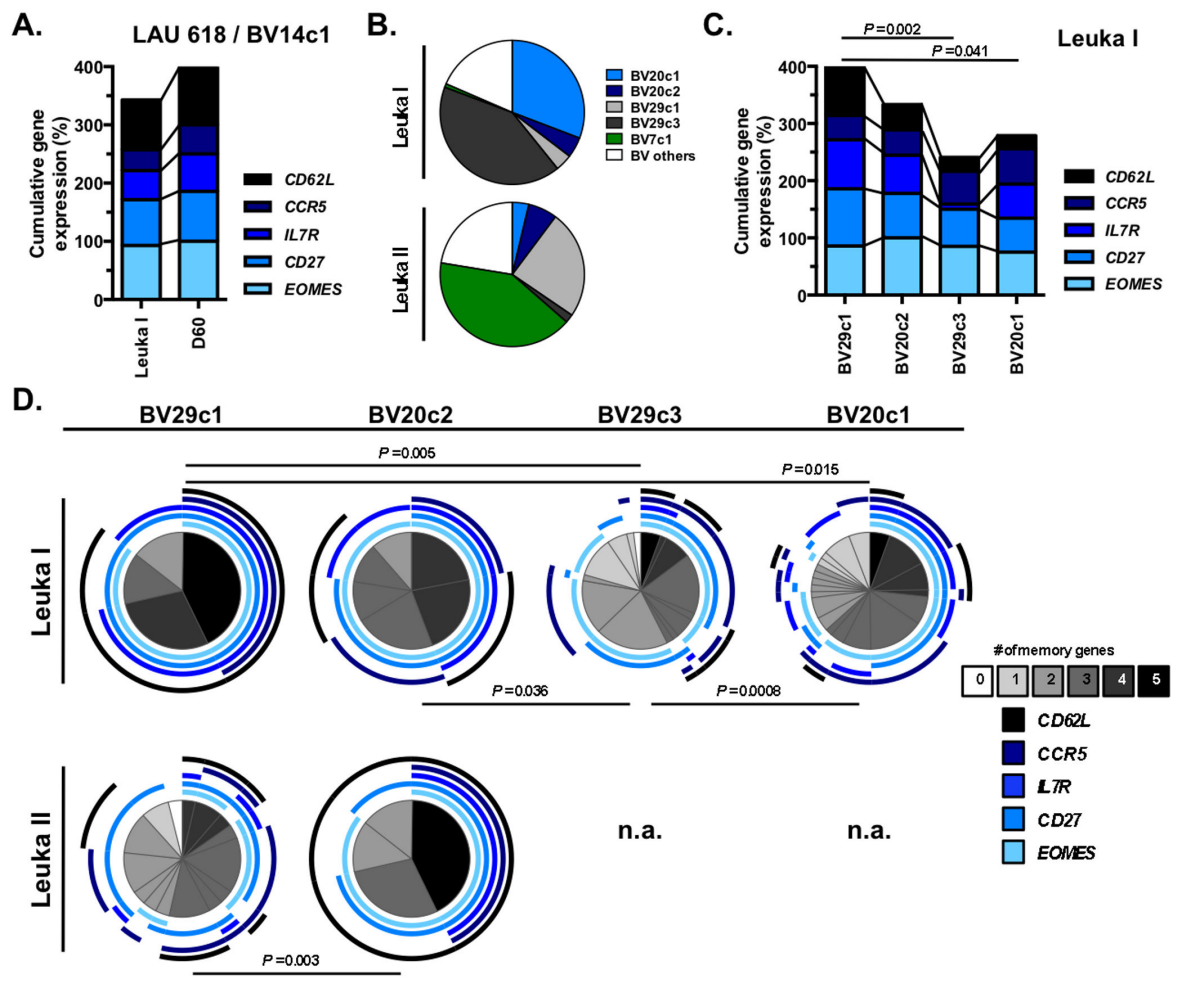
Co-expression of memory-related genes by *ex* *vivo* sorted dominant EBV antigen-specific TCR clonotypes before and following transient lympho-depletion. **A**. Cumulative gene expression analysis of the dominant BV14c1 clonotype from patient LAU 618 at Leuka and post-Leuka (D60) time-points. Expression of memory-associated genes was determined on 5-cell cDNA samples sorted from early-differentiated EM28^pos^ EBV-specific CD8 T cells (n = 28). **B**. Quantification of co-dominant TCR clonotypes from patient LAU 1013 at Leuka I (n = 130) and Leuka II (n = 107) time-points, within the early-differentiated EM28^pos^ subset, shown in frequencies (in %). **C**. Cumulative memory/homing gene expression profile of single cell samples for each of the four dominant clonotypes at Leuka I time-point. Individual EBV-specific CD8 T cell clonotypes from patient LAU 1013 were sorted from the early-differentiated EM28^pos^ subset (n = 94). *P*-values were performed by one-way ANOVA test. **D**. Gene co-expression polyfunctionality was determined on single cell samples representing individual TCR clonotypes from EM28^pos^ EBV-specific CD8 T cell subset at Leuka I (n = 94) and Leuka II (n = 83) from patient LAU 1013. Colors of the pie arcs depict the co-expression of individual memory genes, whereas the color in the pie depicts the number of co-expressed memory-associated genes, as determined by SPICE 5.2. Increased polyfunctional gene co-expression (from 0 to 5) is shown as progressive grey gradients (from white to black). *P*-values of the permutation test are shown.

By combined characterization of cell differentiation and TRBV-CDR3 sequencing, direct *ex vivo* analysis of T cell responses allows longitudinal description of individual clonotypes [13,15,28]. With this strategy, we sought to identify the optimal set of memory-related genes that would best identify T cell clonotypes that survived chemotherapy and persisted over time. High expression of memory/homing associated mRNA transcripts was observed in the dominant BV14c1 clonotype from patient LAU 618 (Figure 6A), which was selected following lympho-depletion and immune reconstitution, 74% and 80% clonotype frequency before and after treatment, respectively (Figure 2C). We then extended our analysis to patient LAU 1013 and performed highly sensitive single cell gene expression analysis on four EBV-specific T cell clonotypes selected based on their persistence following the two rounds of TLD and ACT. From Leuka I to Leuka II, clonotypes BV29c1 increased in frequencies (4% to 24%), clonotype BV20c2 was stable (5% to 7%), whereas clonotypes BV29c3 and BV20c1 decreased in frequencies (42% to 2% and 31% to 4%, respectively) (Figure 6B).

We assessed the memory/homing-associated gene expression profiles of the four T cell clonotypes (Figure 6C) at Leuka I time-point. The persisting BV29c1 clonotype showed significantly enhanced expression of *CD62L/SELL*, *IL7R* and *CD27*, compared to the BV29c3 and BV20c1 clonotypes, which declined after the first round of TLD (*P* = 0.002 and *P* = 0.041 respectively; ANOVA). No major difference in the overall gene expression of effector mediators could be detected (Figure S3). We also analyzed the gene expression polyfunctionality by determining the direct *ex vivo* co-expression patterns of memory/homing-related genes within the co-dominant virus-specific clonotypes in patient LAU 1013 (Figure 6D). Again, the BV29c1 clonotype, which persisted after both lympho-depletion regimens, revealed the highest degree of memory co-expression genes at Leuka I (largely co-expressing four to five memory-associated genes). Conversely, the non-persistent BV29c3 and BV20c1 clonotypes, showing statistically significantly reduced co-expression of mRNAs coding for memory/homing-mediating molecules. Finally, and despite its low clonotypic frequency, the BV20c2 clonotype maintained robust memory/homing-related gene co-expression after the first round of TLD (Leuka II). Of note, complete gene expression profiling of the BV7c1 clonotype, which was selected following several rounds of treatment (Figure 6B; Leuka II), was not possible at Leuka I, as we were unable to identify sufficient individual T cells for the analysis.

Collectively, these results suggest that distinct co-expression patterns of memory-mediating molecules may be involved in the selection and persistence of EBV antigen-specific CD8 T cell clonotypes following transient immune-suppression. Furthermore, this polyfunctional expression profile characterized by high levels of co-expression of memory/homing-related genes may represent a hallmark of T cell clonotypes that are able to survive treatment with cytotoxic drugs and persist over extended periods of time.

## Discussion

The capacity of the EBV-specific immune response to efficiently control viral infection is evidenced by the absence of disease in long-term EBV-infected healthy individuals. Severe immunosuppression following transplantation or HIV infection can result in deficient EBV-specific immunity, which may lead to EBV-associated pathology such as lymphoproliferative disease. Transient lympho-depletion (TLD) induced by non-myeloablative chemotherapy has become of high interest in the field of cancer immunotherapy, as it strongly favors *in vivo* survival and expansion of adoptively transferred cells [31,36-38]. However, the ability of EBV-specific T cells to avoid resurging viral infection and spread following temporary state of immunosuppression remains unknown. 

In the present study, we examined the robustness of the EBV-specific T cell response following TLD in comparison to steady-state conditions over a period of four years. We found that, similarly to the total CD8 population [25], EBV antigen-specific T cells also recovered efficiently following TLD (Figure 1). Although the proportion of differentiated effector-like T cells was variable among the different patients at the leukapheresis time-point, this T cell population uniformly increased following TLD and immune reconstitution. These results are consistent with previous reports describing enhanced T cell differentiation associated with immunosuppressive treatments [39,40], as well as with our formal study showing a decline in naïve CD8 T cell numbers following TLD and a proliferative expansion of differentiated effector-memory T cells [25]. Furthermore, we found no evidence of EBV viral reactivation shortly after ACT and at later time-points (200 days, Figure 1), suggesting that the lympho-depleting regimen was not severe or long enough to cause EBV reactivation. From a clinical point of view, these findings are encouraging as they provide further support for the safety of short-term non-myeloablative chemotherapy regimens [24,25].

Owing to a high fidelity DNA polymerase, viruses belonging to the herpesviridae family are genetically stable [2], thereby allowing the tracking of viral antigen-specific T cells over time. We characterized the TCR beta-chain clonotype repertoire and selection of BMFL1-specific T cells from EBV^pos^ healthy donors under steady-state conditions as well as EBV^pos^ patients undergoing short-term chemotherapy. We found that the T cell clonotype selection and composition of the CD8 T cell response to an immunodominant epitope among patients closely resembled that of healthy individuals (Figure 3). Specifically, both groups displayed a similar degree of clonotype diversity, with selection for dominant T cell clonotypes bearing preferential *TRBV20* and *TRBV29* gene segments. Interestingly, we identified several public TRBV-CDR3 sequences that dominated the T cell clonotype repertoires of healthy donors and patients, including the previously identified archetypal AS01 ubiquitous TCR sequence (*TRBV20-1*/*TRBJ1-2* CSARDGTGNGYTF) [41]. These findings indicate that the T cell clonal composition generated against the BMFL1 epitope in lympho-depleted melanoma patients showed structurally conserved TCR features, highly similar to those found in healthy individuals. The pattern of antigen-specific TCRs has been recently shown to influence the potency of immune responses during SIV/HIV infections [42-44]. In particular, the number of public clonotypes correlated inversely with the viral load after SIV challenges [42,45]. T cell populations with public clonotypes also exhibited a greater functional avidity, which was associated with relative HIV control [43,44], providing new insights into the immune parameters that mediate the spontaneous control of chronic viral infections.

Persistence of herpes virus-specific T cell clonotypes with time has been previously documented in humans [14,21,46]. More recently, using *ex vivo* new generation-sequencing, Klarenbeek and coworkers [23] reported on the stability of the repertoire of anti-viral CD8 T cell responses to CMV and EBV in renal transplanted recipients by demonstrating that nearly all virus-specific clonotypes appearing during the acute phase of infection were maintained over time. Importantly, they showed that the hierarchy of dominance of individual clonotypes was preserved during several years, highlighting the robustness of virus-specific T cell responses. In the present study, we extended on these findings by showing that the *in vivo* BMFL1-specific T cell repertoire was also stable following transient immunosuppression, with the frequent TCR clonotypes remaining dominant despite fluctuations in frequencies of low-dominant clonotypes (Figure 2). The small number of patients (n = 5) may represent a limitation of the current study, which primarily reflects the small number of patients undergoing phase I clinical trials using lympho-depleting chemotherapy. Nevertheless, this cohort of EBV^pos^ carriers represents a unique opportunity to study the EBV-specific responses during transient immunological perturbations, in well-defined clinical settings, and not only over time under steady-state conditions.

The persistence of the EBV antigen-specific clonal repertoire following TLD and immune reconstitution represents an interesting observation as more pronounced fluctuations might have been expected. These include *de novo* T cell priming and thus the emergence of novel TCR clonotypes, and/or loss of (less frequent) clonotypes, presumably because of increased competition for survival factors. Moreover, the immune reconstitution period following TLD is generally associated with a skewing of the global CD8 T cell repertoire, in part due to early preferential expansion of antigen-primed T cells, followed by the slow repopulation with new thymic emigrants that eventually re-establish a high TCR repertoire diversity (reviewed in [47]). Furthermore, a recent analysis of the TCR repertoire following stem cell transplantation showed that the dynamics of recovery of the TCR repertoire diversity largely depends on the source of donor cells, with cord-blood cells being the most efficient, whereas T cell-depleted peripheral blood stem cells the least efficient at rapidly establishing TCR diversity [48]. Since the source of our re-infused cells was autologous unmodified PBMCs, we did not expect this to have a major impact on the overall TCR diversity following ACT. Specifically, our results did not show evidence of TCR repertoire significant narrowing following transient lympho-depletion and immune reconstitution since the majority of dominant EBV-specific T cell clonotypes survived efficiently to TLD.

Narrowing of the CD8 TCR repertoire can be observed in old age and/or by CMV infection, leading to increased susceptibility to infection [49]. In the present study, the CMV positive status did not correlate with a more restricted EBV-specific TCR repertoire (e.g. patients LAU 618, LAU 672 and LAU1013), however the most restricted EBV-specific TCR repertoires before treatment were observed for the two oldest patients in our cohort (LAU 1144 and LAU 618). Nevertheless, despite the advanced age and restricted TCR repertoires, total and EBV-specific CD8 T lymphocyte counts recovered well following chemotherapy and adoptive cell transfer. 

Technological advancements in the characterization of T lymphocyte responses reveal an unforeseen degree of heterogeneity of the antigen-primed T cell pool. Moreover, the level of polyfunctionality manifested by individual T cells can often be under-estimated when studying defined cellular populations by flow cytometry. In contrast, single cell analyses allow the accurate characterization of the functional phenotype of individual T cells. Our group and others have developed sophisticated laboratory techniques to analyze the gene expression signatures in individual T cells directly *ex vivo* [28,50,51]. Such analyses provide significant advantages in the context of characterizing antigen-specific immune responses. Single cell gene expression profiling can complement flow cytometry data by expending the number of genes/populations markers used to define specific subpopulations, thereby offering a more detailed characterization of memory or effector subsets. For instance, this technique has proven useful in identifying previously unrecognized subsets of CD8 T cells differentially induced by various vaccines [51] as well as revealing important differences between the polyfunctional gene-expression profiles of T cells induced after vaccination with two closely related tumor antigenic peptides [13,28]. In these studies, gene expression analysis was extensively validated by comparisons with expression at the protein level using multi-parameter flow cytometry [13].

Direct *ex vivo* single T cell gene expression analyses allows the accurate determination of the genes expressed by (multiple representatives of) individual TCR clonotypes. As the generation of clonotypic-specific mAbs to identify such individual clonotypes by flow cytometry still remains technically challenging, the identification of single T cell clonotypes is currently most reliably performed at the gene expression level. We have previously shown strong concordance between TCR repertoire analysis performed on *ex vivo* sorted cells and *in vitro* generated T cell clones [28], as well as between independently performed *ex vivo* single-cell sorted experiments (Figure S2). These data were obtained from *ex vivo* sorted single cells, representing the equivalent of 300 to 450 cells per healthy donor or melanoma patient. They indicate the high sensitivity of our molecularly based approach for assessing gene expression in individual antigen-specific T cell clonotypes directly *ex vivo*, and allow the fine characterization of the parameters involved in memory T cell formation and maintenance over time (Figures 4 to 6). 

ACT studies performed in mice show that T cell populations expressing L-selectin (CD62L) and IL-7Ra display improved survival compared with subsets lacking the expression of these markers [52,53]. Results from clinical trials involving ACT in humans suggest that early-differentiated central memory T cells have a survival advantage *in vivo* due to their enhanced proliferative and self-renewal capacities after adoptive transfer [38,54,55]. We recently reported that under steady-state conditions, the early-differentiated EM28^pos^ EBV-specific T cells show high co-expression of memory/homing-related genes and relatively infrequent effector-gene expression [28]. Thus, we hypothesized that the persisting anti-viral CD8 T cell clonotypes might exhibit a memory-associated phenotype, which would favor their persistence following TLD. By profiling the expression of genes related to memory/homing and effector T cell properties, we found that persisting clonotypes (such as the BV29c1 clonotype from patient LAU 1013 and BV14c1 from patient LAU 618) showed specific and robust gene co-expression of *CD27*, *IL7R*, and *CD62L/SELL* prior to treatment (Figure 6). Conversely, we observed significant differences in the memory gene expression profiling among two other dominant but non-persisting T cell clonotypes (BV29c3 and BV20c1 from patient LAU 1013). Although this in-depth analysis was only possible on few clonotypes, our data suggest that a polyfunctional memory/homing gene co-expression profile before TLD may be predictive of TCR clonotypes with improved survival during the immune reconstitution period. These results are in line with the concept that polyfunctional viral-specific T cells have also been observed in responses to the persistent and well-controlled CMV viral infection [28], as well as within long-term non-progressors of HIV-infected individuals [56]. Identifying specific gene expression profiles involved in the survival and persistence of antigen-specific T lymphocytes over time will help us in understanding correlates of immune protection and are essential to promote therapeutic immune intervention like vaccination or adoptive T cell therapy. 

CD27 expression on T cells is associated with resistance to apoptosis, high IL-2 production, and increased T cell expansion [57], while IL-7Ra is a hallmark of primed CD8 T cells with the capacity to develop into long-lived memory cells [52,58]. As a result of cytoreduction following lympho-depleting chemotherapy, there is an increase in serum levels of IL-7 and IL-15 due to diminished competition for these cytokines [47]. As such, T cells expressing IL-7 and IL-15 receptors are expected to have survival and proliferative advantages during the immune reconstitution period. Our group previously reported that serum levels of IL-7 were detectable in these patients before TLD and ACT, and remained elevated for long periods of time following treatment [25]. Here, we show that despite the induction of T cell differentiation following TLD and immune recovery, anti-viral persistent T cell clonotypes retained relative high gene expression of *IL7R* and *CD27* even after two rounds of lympho-depletion treatment. Collectively, these data provide further support for the crucial role of IL-7 in memory T cell homeostasis and persistence, and the potential clinical benefits of including IL-7 administration in future vaccination and ACT strategies.

In conclusion, detailed analyses of phenotype, gene expression and TCR clonal repertoire of EBV antigen-specific CD8 T cells in healthy donors and melanoma patients revealed that the anti-viral specific clonotypes persisted over time and showed the ability to resist cytotoxic chemotherapy. Importantly, the *in vivo* setting studied here differs from the well-established allogeneic bone marrow transplantation in which the immunological perturbations are likely more profound. It also differs from the phase I clinical trials, which involve the same non-myeloablative lympho-depleting chemotherapy regimens, but with the transfer of either tumor-infiltrating lymphocyte cultures [31] or autologous CD3 T lymphocytes engineered with antigen-specific TCRs [59,60]. Future studies are needed to characterize the impact of these *in vivo* settings on the dynamics of anti-viral T cell responses. Furthermore, direct *ex vivo* high-resolution molecular characterization of individual T cells at the clonotype level, as shown here, provides enhanced insights in the processes shaping the *in vivo* long-term survival of anti-viral specific T cells. 

## Supporting Information

Figure S1 *Exvivo* phenotype analysis of HLA-A*0201/BMFL1-specific CD8 T cells in melanoma patients before and after transient lympho-depletion. Representative *ex* *vivo* flow cytometry analysis of EBV antigen-specific CD8 T cells in patient LAU672 at time-points before (Leuka) and after TLC (post-ACT). Total CD8 T cells (R1 gated top panels) and EBV-multimer^**pos**^ T cells (HLA-A2/BMLF1 defined as GLC/A2; R2 gated bottom panels) were characterized for the cell surface expression of CCR7 and CD45RA (middle panels). Double staining for CD45RA and CD28 is shown for CCR7 negative gated populations. Quadrant percentages are depicted for each subset.
(TIF)

Figure S2 **Reproducibility of TCR BV clonotype analysis.** **A**. Positive correlation of clonotype frequencies obtained between direct *ex* *vivo* 5-cell sample sorting (n = 162) and *in* *vitro* generated single-cell cloning (n = 676) by Spearman’s correlation. Plot shows all TCR clonotypes identified in blood samples at leukapheresis time-points from four melanoma patients with inset showing the correlation between TCR clonotypes with frequencies below 20%. **B**. Analysis of the TCR repertoire diversity of EBV-specific CD8 T cells from patient LAU 1013 at leuka II (n = 107) obtained from single-cell samples directly *ex* *vivo* sorted from two separate experiments. Each dominant clonotype is indicated and color-coded. Non-dominant clonotypes are designed as “BV others” and are composed of non-clonotypic sequences.
(TIF)

Figure S3 **Co-expression of effector-related genes by dominant EBV antigen-specific TCR clonotypes before and following transient lympho-depletion.** **A**. Cumulative effector-gene expression profile of single cell samples for each of the four dominant clonotypes at Leuka I time-point. Individual EBV antigen-specific CD8 T cell clonotypes from patient LAU 1013 were sorted from the early-differentiated EM28^pos^ subset (n = 94). **B**. Gene co-expression polyfunctionality was determined on single cell samples representing individual TCR clonotypes from EM28^pos^ EBV antigen-specific CD8 T cell subset at Leuka I (n = 94) and Leuka II (n = 83) from patient LAU 1013. Colors of the pie arcs depict the co-expression of individual effector genes (*PRF1*, *KLRD1/CD94*, *IFNG* and *GZMB*), whereas the color in the pie depicts the number of co-expressed effector-related genes, as determined by SPICE 5.2. Increased polyfunctional gene co-expression (from 0 to 4) is shown as progressive grey gradients (from white to black). *P*-values of the permutation test are shown.
(TIF)
