# Supplementary figures and images for: Persistence of EBV Antigen-Specific CD8 T Cell Clonotypes during Homeostatic Immune Reconstitution in Cancer Patients
Source: PLoS One. 2013 Oct 25;8(10):e78686. doi: 10.1371/journal.pone.0078686 (PMC3808305; doi:10.1371/journal.pone.0078686)

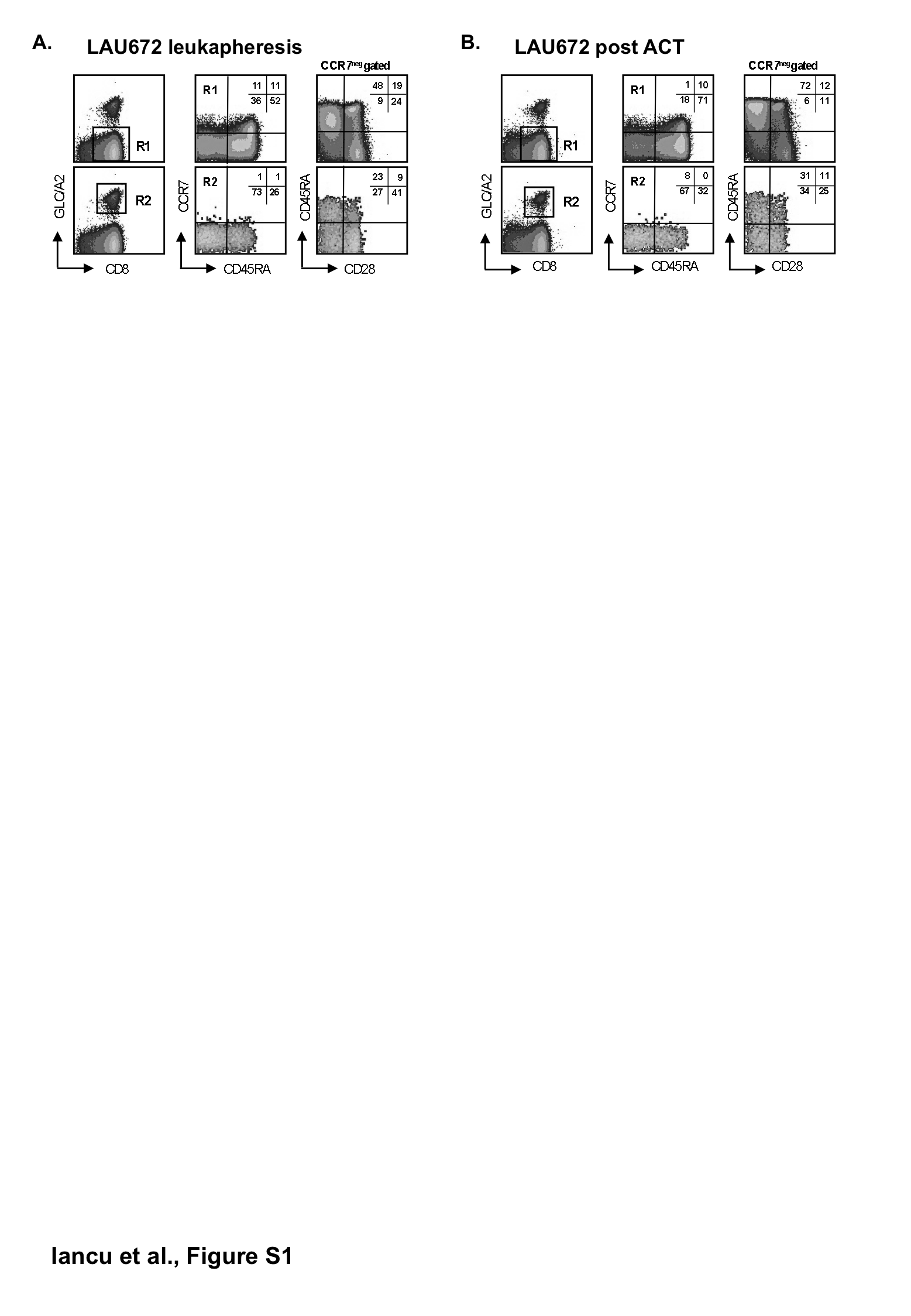

Supplement: Figure S1 — Exvivo phenotype analysis of HLA-A*0201/BMFL1-specific CD8 T cells in melanoma patients before and after transient lympho-depletion. Representative ex vivo flow cytometry analysis of EBV antigen-specific CD8 T cells in patient LAU672 at time-points before (Leuka) and after TLC (post-ACT). Total CD8 T cells (R1 gated top panels) and EBV-multimerpos T cells (HLA-A2/BMLF1 defined as GLC/A2; R2 gated bottom panels) were characterized for the cell surface expression of CCR7 and CD45RA (middle panels). Double staining for CD45RA and CD28 is shown for CCR7 negative gated populations. Quadrant percentages are depicted for each subset. (TIF) [file pone.0078686.s001.tif]

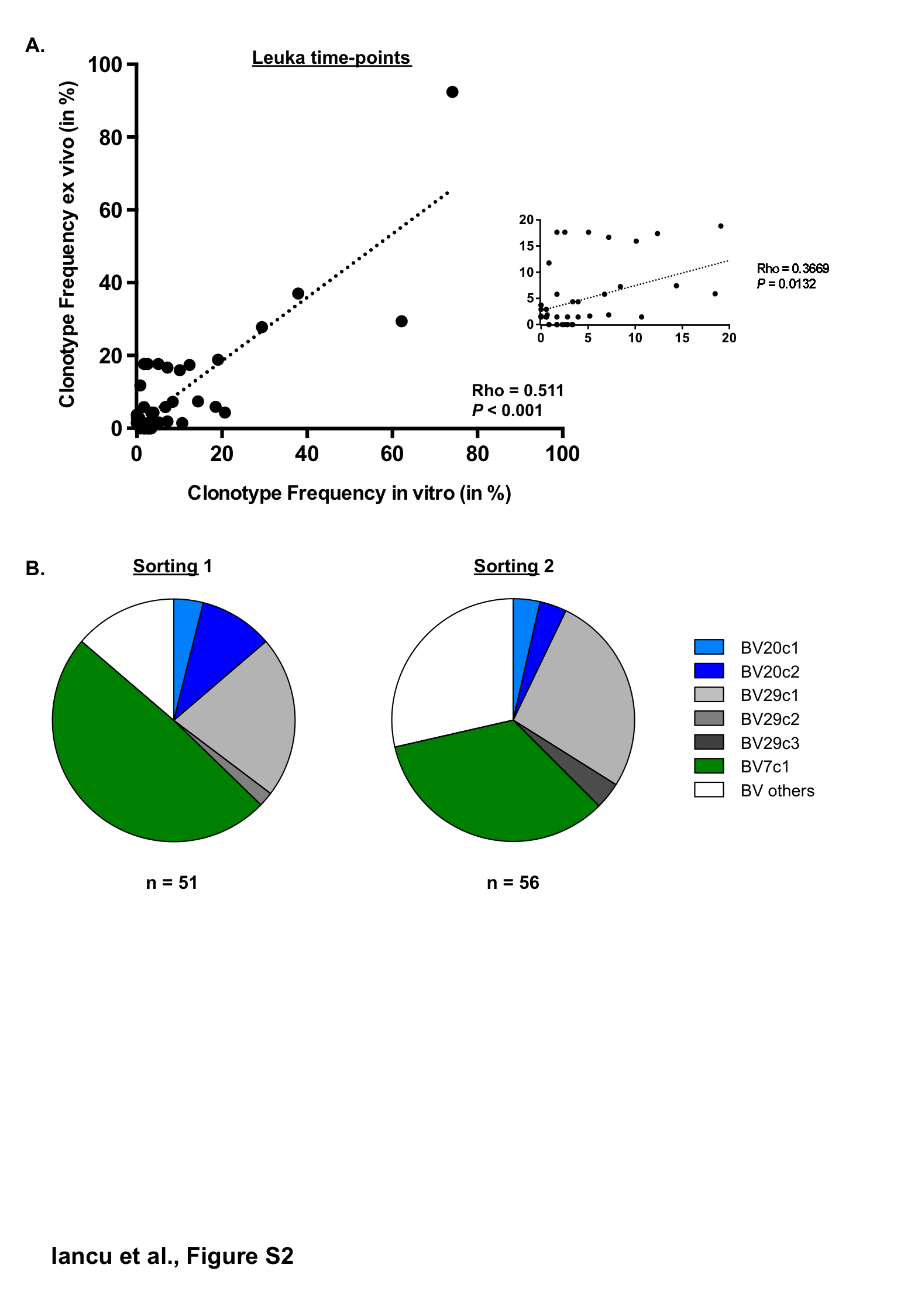

Supplement: Figure S2 — Reproducibility of TCR BV clonotype analysis. A. Positive correlation of clonotype frequencies obtained between direct ex vivo 5-cell sample sorting (n = 162) and in vitro generated single-cell cloning (n = 676) by Spearman’s correlation. Plot shows all TCR clonotypes identified in blood samples at leukapheresis time-points from four melanoma patients with inset showing the correlation between TCR clonotypes with frequencies below 20%. B. Analysis of the TCR repertoire diversity of EBV-specific CD8 T cells from patient LAU 1013 at leuka II (n = 107) obtained from single-cell samples directly ex vivo sorted from two separate experiments. Each dominant clonotype is indicated and color-coded. Non-dominant clonotypes are designed as “BV others” and are composed of non-clonotypic sequences. (TIF) [file pone.0078686.s002.tif]

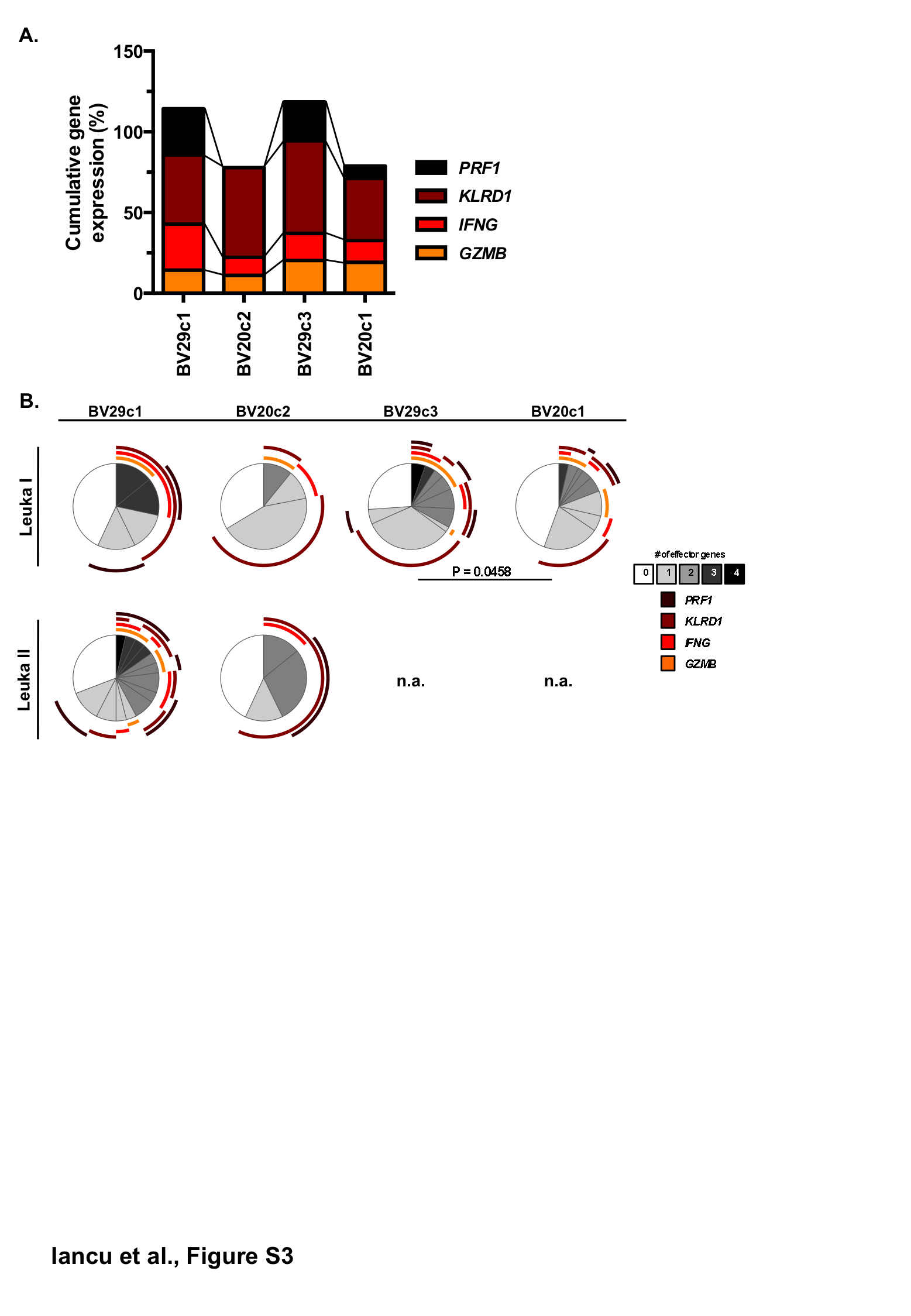

Supplement: Figure S3 — Co-expression of effector-related genes by dominant EBV antigen-specific TCR clonotypes before and following transient lympho-depletion. A. Cumulative effector-gene expression profile of single cell samples for each of the four dominant clonotypes at Leuka I time-point. Individual EBV antigen-specific CD8 T cell clonotypes from patient LAU 1013 were sorted from the early-differentiated EM28pos subset (n = 94). B. Gene co-expression polyfunctionality was determined on single cell samples representing individual TCR clonotypes from EM28pos EBV antigen-specific CD8 T cell subset at Leuka I (n = 94) and Leuka II (n = 83) from patient LAU 1013. Colors of the pie arcs depict the co-expression of individual effector genes (PRF1, KLRD1/CD94, IFNG and GZMB), whereas the color in the pie depicts the number of co-expressed effector-related genes, as determined by SPICE 5.2. Increased polyfunctional gene co-expression (from 0 to 4) is shown as progressive grey gradients (from white to black). P-values of the permutation test are shown. (TIF) [file pone.0078686.s003.tif]
